# Nonoperative treatment of insertional Achilles tendinopathy: a systematic review

**DOI:** 10.1186/s13018-021-02370-0

**Published:** 2021-03-30

**Authors:** Xiaosong Zhi, Xinyuan Liu, Jing Han, Yang Xiang, Helin Wu, Shijun Wei, Feng Xu

**Affiliations:** 1 Department of Orthopaedics, General Hospital of Central Theater Command (Wuhan General Hospital of Guangzhou Command, previously), NO. 627, Wuluo Road, Wuhan, 430030 Hubei Province P. R. China; 2 Department of Emergency, Taikang Tongji (Wuhan) Hospital, Wuhan, Hubei Province P. R. China; 3 Graduate School of Wuhan University of Science and Technology, Wuhan, Hubei Province P. R. China; 4 The First Clinical Medical School Of Southern Medical University, Guangzhou, Guangdong Province P. R. China

**Keywords:** Insertional Achilles tendinopathy, Nonoperative treatment, Systematic review

## Abstract

**Background:**

Insertional Achilles tendinopathy is difficult to manage, and there is no definite consensus on which nonoperative treatment is superior over the others. We aim to provide a clear summary of the best available evidence for nonoperative treatment specific to insertional Achilles tendinopathy.

**Methods:**

Literatures were searched in PubMed, Embase, and Web of Science databases from inception to October 2020. The results were evaluated independently by two reviewers and assessed against the inclusion/exclusion criteria. All included articles were assessed for methodological quality, and study characteristics were extracted.

**Results:**

Twenty-three studies (containing 35 groups) were eligible for the final review. The treatments included eccentric training, extracorporeal shockwave therapy (ESWT), injections, and combined treatment. Visual analog scale (VAS), Victorian Institute of Sport Assessment-Achilles questionnaire, AOFAS, satisfaction rate, and other scales were used to assess the clinical outcome.

**Conclusion:**

Current evidence for nonoperative treatment specific for insertional Achilles tendinopathy favors ESWT or the combined treatment of ESWT plus eccentric exercises.

**Supplementary Information:**

The online version contains supplementary material available at 10.1186/s13018-021-02370-0.

## Introduction

Insertional Achilles tendinopathy is located at the insertion of the Achilles tendon onto the calcaneus, possibly with the formation of bone spurs and calcifications in the tendon proper at the insertion site. Patients complain of pain, stiffness, and sometimes (a solid) swelling. On physical examination, the tendon insertion is painful. A swelling may be visible and a bony spur may be palpable [1]. The incidence of Achilles tendon pain is approximately 6% in the general population, and 24% of the people suffering from heel pain were diagnosed as insertional Achilles tendinopathy (IAT) [2, 3]. The incidence of IAT increases with age and is significantly higher in patients with metabolic diseases (e.g., diabetes mellitus, hypercholesterolemia, and hypothyroidism) [4]. However, the exact etiology and pathogenesis remain unclear.

Usually, nonoperative treatment, including eccentric exercise, extracorporeal shock wave therapy (ESWT), NSAIDs, orthotics, laser therapy, platelet-rich plasma injections (PRP), corticosteroid injections, and sclerosant injection, are the primary treatment for Achilles tendinopathy and are mostly effective especially in mid-portion/non-insertional Achilles tendinopathy (pathology at 2–6 cm proximal to Achilles insertion). However, it is widely considered that IAT is a distinct clinical entity [5], and non-surgical interventions in IAT have not shown expectative clinical outcomes as good as the same treatment in mid-portion Achilles tendinopathy [6]. Furthermore, there is no definite consensus on which nonoperative treatment is superior over the others. So, we performed this systematic review to analyze the effectiveness of different currently used nonoperative therapy for IAT.

## Method

This systematic review was carried out following the Preferred Reporting Items for Systematic Reviews and Meta-Analyses (PRISMA) guidelines [7, 8] during the stages of design, analysis, and reporting.

### Search strategy

Literatures were searched in PubMed, Embase, and Web of Science databases from inception to October 2020. The search items were as follows: (insertional OR insertion) AND (tendinopathy OR tendinitis OR tendinosis OR enthesitis or enthesopathy) AND Achilles. Additional potential literatures were obtained by searching the reference list of the identified full-text articles.

### Inclusion and exclusion criteria

An article included should meet all the following criteria: (1) randomized controlled trial (RCT), non-randomized comparative study, prospective cohort, retrospective cohort study, or case series; (2) patients with insertional Achilles tendinopathy were clinically diagnosed with or without imaging confirmation; (3) nonoperative treatment was applied and clearly described; and (4) reporting the outcome regarding pain or function.

A study was excluded if it met one of these criteria: (1) review articles, meta-analysis, case reports, editorial, surgical articles, cadaveric studies, or animal experiments; (2) treatment only on mid-portion or non-insertional tendinopathy; (3) treatment on insertional Achilles tendinopathy but the data could not be extracted separately; and (4) non-English articles.

### Study selection and data extraction

Two independent authors reviewed all studies by reading titles, abstracts, and full-texts according to the inclusion and exclusion criteria mentioned above. The critical information in the final selected studies were independently extracted by two authors. Any discrepancy was resolved by discussing it until a consensus was reached. The extracted data included the last name of the first author, publication year, level of evidence, study design, publication country, sample size, activity level, mean age, duration of symptoms, diagnosis method, interventions, follow-up time, and outcome.

### Quality assessment

All studies were assessed with the level of evidence [9]. The risk of bias of randomized controlled trials (RCTs) was evaluated using the Cochrane bias tool (https://handbook-5-1.cochrane.org/), which covered six domains: random sequence generation and allocation concealment (selection bias), blinding of participants (performance bias), blinding of outcome assessment (detection bias), incomplete outcome data (attrition bias), selective reporting (reporting bias), and other bias. Each item was categorized as “high”, “low,” or “unclear.” The methodological quality of non-randomized studies was assessed by methodological index for non-randomized studies (MINORS) covering 12 items with a total score of 24 for comparative studies, in which the first 8 items with a total score of 16 for non-comparative studies [10]. Any discrepancy was resolved by discussing or consulting an expert investigator until a consensus was reached.

### Statistical analyses

The data is presented as weighted means and summed percentages. Statistical analyses from the included studies were used in determining statistical significance of the data. *P* values are extracted from the original studies or calculated from the reported data. STATA version 12.0 (StataCorp LP, College Station, TX, USA) was used for the whole analysis. Statistical significance was set at *P* < .05.

## Results

### Literature search

**Fig. 1:**
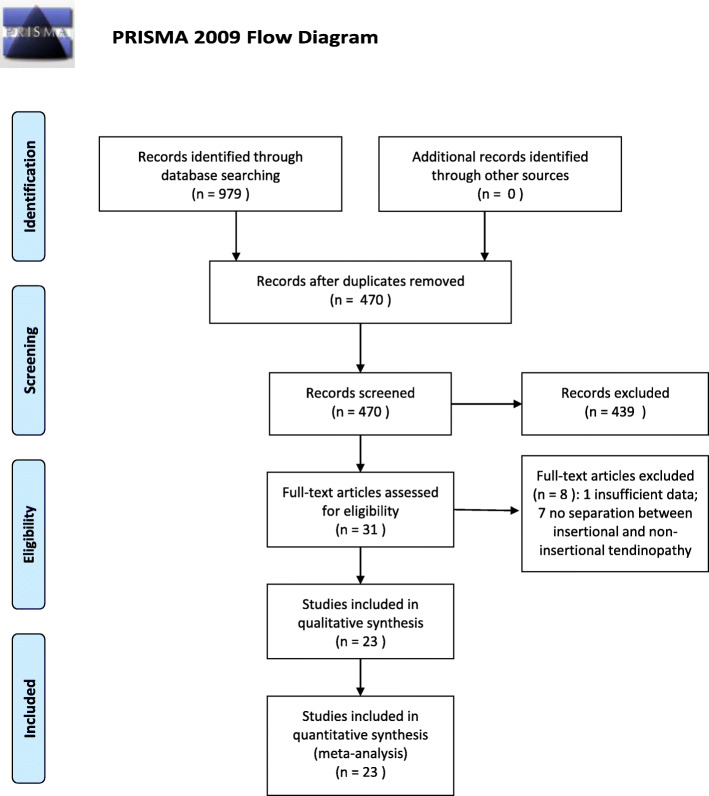
Flowchart of the literature selection

The search of PubMed, Embase, Web of Science database, and additional search finally yielded 470 records after duplicates were removed. The studies were screened by reviewing abstracts and full-texts according to the inclusion and exclusion criteria, and 23 studies [6, 11–32] (containing 35 groups) were eligible for the final review, including 11 comparative studies (6 RCTs, 5 non-randomized comparative studies) and 12 non-comparative studies. The studies were published from 2003 to 2020. Of those, 3 studies were conducted in Sweden, 4 in the USA, 2 in Germany, 1 in Canada, 1 in Australia, 5 in Italy, 3 in the UK, 2 in China, 1 in Brazil, and 1 in Thailand. The detailed information is shown in Fig. 1 and Table 1. This study followed the PRISMA 2009 checklist as provided in Additional file 1.

**Table 1:** Characteristics of included studies and population

No.	Study	Year	LOE	Study design	Country	No. of patients (tendons)	Activity level	Age (year)	Duration of symptoms (month)	Diagnosis
1	Fahlström et al.	2003	4	Prospective	Sweden	30 (31)	Recreational	37.9	32	CE, US, MRI, X-ray
2	Öhberg et al.	2003	4	Pilot study	Sweden	11 (11)	Mixed	44	29	CE, US
3	Costantino et al.	2005	3	Retrospective	Italy	Group A 5 (5)	Professional	32.7	≥ 2	CE, US
						Group B 5 (5)	Professional	32.7	≥ 2	
						Group C 5 (5)	Professional	32.7	≥ 2	
4	Furia et al.	2006	3	Retrospective	USA	Group A 35 (35)	Mixed	50	19.9	CE, MRI, X-ray
						Group B 33 (33)	Mixed	52.6	16.8	
5	Knobloch et al.	2007	4	Prospective	Germany	10 (10)	Recreational	NR	≥ 3	CE, US
6	Rompe et al.	2008	1	RCT	Germany	25 (30)	Mixed	39.2	24.8	CE, US, X-ray
						25 (31)	Mixed	40.4	26.3	
7	Jonsson et al.	2008	4	Pilot study	Sweden	27 (34)	Recreational	53.4	26.5	CE, US
8	Ryan et al.	2010	4	Case series	Canada	NR (22)	NR	NR	≥ 6	CE, US
9	Verrall et al.	2011	4	Retrospective	Australia	14 (14)	Professional	NR	4.5	CE
10	Saxena et al.	2011	2	Prospective	USA	NR (19)	NR	54.3	NR	CE, MRI, X-ray
11	Notarnicola et al.	2012	1	RCT	Italy	Group A 32 (32)	NR	NR	6	CE, US, MRI, X-ray
						Group B 32 (32)	NR	NR	6	
12	Notarnicola et al.	2013	2	RCT	Italy	Group A 30 (30)	NR	57.5	≥ 6	CE, US, MRI, X-ray
						Group B 30 (30)	NR	59.5	≥ 6	
13	Kedia et al.	2014	2	RCT	USA	Group A 16 (19)	Mixed	51.7	18.5	CE
						Group B 20 (20)	Mixed	55.3	18.3	
14	McCormack et al.	2016	2	RCT	USA	Group A 6 (6)	NR	53.3	21.9	CE
						Group B 6 (6)	NR	53.9	20.8	
15	Taylor et al.	2016	4	Prospective	UK	12 (12)	NR	54	42	CE, US
16	Pavone et al.	2016	4	Case series	Italy	40 (40)	NR	41	≥ 3	CE, X-ray
17	Wu et al.	2016	3	Retrospective	China	Group A 37 (37)	NR	37.6	≥ 6	CE, X-ray
						Group B 30 (30)	NR	35.8	≥ 6	
18	Erroi et al.	2017	3	Retrospective	Italy	Group A 24 (24)	Mixed	53.2	13.7	CE, US
						Group B 21 (21)	Mixed	47.7	14.3	
19	Maffulli et al.	2018	4	Case series	UK	80 (80)	NR	53.4	NR	CE
20	Wheeler et al.	2019	4	Case series	UK	30 (30)	NR	55.4	21	CE
21	Mansur et al.	2019	3	Prospective	Brazil	19 (19)	NR	51	NR	CE, US, X-ray
22	Pinitkwamdee et al.	2020	1	RCT	Thailand	Group A 16 (22)	Mixed	61.4	7.5	CE,X-ray
						Group B 15 (16)	Mixed	56.5	12	
23	Zhang et al.	2020	3	Retrospective	China	Group A 16 (16)	Sports-active	31	≥ 3	CE, US, MRI
						Group B 17 (17)	Nonsports-active	37	≥ 3	

*Abbreviations*: *LOE* level of evidence, *No.* number, *RCT* randomized controlled trial, *NR* not reported, *CE* clinical examination, *US* ultrasound, *MRI* magnetic resonance imaging

### Population characteristics

The available data of numbers of patients (tendons) extracted from the included studies are shown in Table 1. Patients with bilateral symptoms were enrolled in 5 studies. Two studies only reported the number of affected tendons, but the number of patients was not given. The symptom duration time of patients were all above 2 months (not reported in 3 studies). Patients in 5 studies were diagnosed only by clinical examination (CE); in 6 studies by CE and ultrasound (US); in 3 studies by CE and X-ray; in 2 studies by CE, US, and X-ray; in 2 studies by CE, MRI, and X-ray; in 1 study by CE, US, and MRI; and in 3 studies by CE, US, MRI, and X-ray. All patients had follow-ups at least 3 months after specific treatments.

### Quality assessment

The detailed information of quality assessment is shown in Table 2 and Table 3. Seventeen studies were non-randomized studies, and the methodological quality was assessed by MINORS. Five of them were comparative studies, and the scores ranged from 15 to 22. The remaining 12 were non-comparative studies, and the scores ranged from 10 to 12. Six studies were RCTs, and the risk of bias was evaluated by the Cochrane bias tool. For “Other bias” assessment, 4 studies were classed as “high risk” (3 studies pooled both unilateral and bilateral cases, 1 study lacked of randomization on the baseline). The other items of the included RCTs were classed as “low risk” or “unclear.”

**Table 2:** MINORS of non-randomized studies

Studies	Study design	MINORS
Fahlström et al. 2003 [6]	Non-comparative	12
Öhberg et al. 2003 [11]	Non-comparative	11
Costantino et al. 2005 [12]	Comparative	18
Furia et al. 2006 [13]	Comparative	20
Knobloch et al. 2007 [14]	Non-comparative	12
Jonsson et al. 2008 [16]	Non-comparative	12
Ryan et al. 2010 [17]	Non-comparative	12
Verrall et al. 2011 [18]	Non-comparative	10
Saxena et al. 2011 [19]	Non-comparative	12
Taylor et al. 2016 [24]	Non-comparative	10
Pavone et al. 2016 [25]	Non-comparative	12
Wu et al. 2016 [26]	Comparative	22
Erroi et al. 2017 [27]	Comparative	18
Maffulli et al. 2018 [28]	Non-comparative	12
Wheeler et al. 2019 [29]	Non-comparative	12
Mansur et al. 2019 [30]	Non-comparative	12
Zhang et al. 2020 [32]	Comparative	15

**Table 3:** Summary of risk of bias of randomized studies

Studies	Random sequence generation (selection bias)	Allocation concealment (selection bias)	Blinding of participants (performance bias)	Blinding of outcome assessment (detection bias)	Incomplete outcome (attrition bias)	Selective reporting (reporting bias)	Other bias
Rompe et al. 2008 [15]	Low	Low	Unclear	Low	Low	Unclear	High
Notarnicola et al. 2012 [20]	Low	Unclear	Low	Low	Low	Unclear	High
Notarnicola et al. 2013	Unclear	Unclear	Low	Unclear	Low	Unclear	Unclear
Kedia et al. 2014 [22]	Low	Low	Unclear	Low	Low	Unclear	High
McCormack et al. 2016 [23]	Low	Low	Unclear	Unclear	Low	Unclear	Unclear
Pinitkwamdee et al. 2020 [31]	Low	Low	Low	Low	Low	Unclear	High

### Eccentric training

There were 7 groups, a total of 128 patients (144 tendons), receiving eccentric training treatment (Table 4). Of those, 6 groups performed a 12-week daily eccentric training regimen, and 1 group was treated with a 6-week eccentric stretching regimen. The training protocol also varied between studies: 5 groups required full-range eccentric exercises (heel lower than forefoot), the other 2 groups performed floor-level eccentric training. Two groups failed in previous nonoperative management (including injection of a local anesthetic and/or a corticosteroid, physiotherapy and/or use of orthotics or a heel lift) before eccentric training. One group received combined eccentric training and conventional physical therapy including gastrocnemius, soleus, hamstring stretches, and ice massage on the Achilles tendon twice a day. With regard to the outcome, a 10-point visual analog scale (VAS) or numerical pain scale was reported in 7 studies. After combining the results, the weighted mean of declined value of the pain scale was 2.83. In 2 groups who failed in the previous nonoperative management, the mean declined value was 1.95 versus 3.48 in the other 5 groups. The patient satisfaction after eccentric training was documented in 5 studies. Overall, 47 of 103 (45.6%) patients thought the outcome was excellent/good, while the others considered it as fair/poor. And the satisfactory rate of 2 groups (failed in previous treatments) was 30.4% vs. 63.8% in the other 3 groups. A Victorian Institute of Sport Assessment-Achilles questionnaire (VISA-A) was finished in 2 studies. The mean value increased from 50.6 to 64.

**Table 4:** Summary of ECC training and outcomes in identified studies

Study	Year	Intervention	LOE	Previous treatment	Follow-up (month)	Evaluation and outcome	Significance	Overall
Fahlström	2003	ECC training (full range): 12 weeks	4	Failed	3	VAS: 7.6 to 5.5	n.s.	VAS: − 2.83 VISA-A: 50.6 to 64 Satisfaction: 45.6% (47/103)
						Satisfaction: 32.2%		
Knobloch	2007	ECC training (full range): 12 weeks	4	None	3	VAS: 6 to 3.2	*	
Rompe	2008	ECC training (full range): 12 weeks	1	Failed	4	VAS: 6.8 to 5.0	*	
						VISA-A: 52.7 to 63.4	*	
						Satisfaction: 28%		
Jonsson	2008	ECC training (floor level): 12 weeks	4	NR	4	VAS: 7.2 to 3.3	*	
						Satisfaction: 66.7%		
Verrall	2011	ECC training (full range): 6 weeks	4	NR	3	VAS: 7.3 to 3.0	*	
						Satisfaction: 50%		
Kedia	2014	ECC (full range)+strengthening training: 12 weeks	2	NR	3	VAS: − 2.19	*	
						SF-36 (bodily pain): 16.22	*	
						SF-36: 9.78	n.s.	
						FAOQ: − 0.73	*	
McCormack	2016	ECC training (floor level): 12 weeks	2	None	13	VAS: 5.4 to 1	*	
						VISA-A: 40.2 to 67	*	
						Satisfaction: 83.3%		

*n.s.* no significance

*P < 0.05

### Extracorporeal shockwave therapy

Patients in 12 groups were treated with either exclusively extracorporeal shockwave therapy (ESWT) or ESWT combined with other nonoperative therapy (without eccentric training) (Table 5). Eleven of the 12 groups were applied with low-energy ESWT (shockwave energy < 0.28 mJ/mm^2^), 1 of them received high-energy ESWT (shockwave energy > 0.6 mJ/mm^2^) [24]. Besides, 7 of the 12 groups had a failure of previous nonoperative therapy. One group was treated with ESWT and additional arginine supplement. Another group received both ESWT and other conventional treatment but without eccentric training. One group was diagnosed with IAT with a Haglund’s deformity. VAS scale was evaluated in 9 of the 12 groups at the final follow-up. The weighted mean of decreased VAS in 8 groups was 4.49 points. The change in the range of VAS in sub-groups showed as 5.10 (high energy) vs. 4.40 (low energy) and 4.15 (failure in previous therapy) vs. 5.12 (no previous therapy). VISA-A was assessed in 7 of the 12 groups, and the mean value increased from 47.5 to 76.2 in combined results. AOFAS was recorded in 2 groups, and the mean value increased from 68.2 to 84.5. Five groups were evaluated with satisfactory results, and 101 out of 137 (73.7%) patients were satisfied with the outcome.

**Table 5:** Summary of ESWT and outcomes in identified studies

Study	Year	Intervention	LOE	Previous treatment	Follow-up (month)	Evaluation and outcome	Significance	Overall
Furia	2006	ESWT (high energy)	3	Failed	12	VAS: 7.9 to 2.8	*	VAS: − 4.49 VISA-A: 47.5 to 76.2 Satisfaction: 73.7% (101/137) AOFAS: 68.2 to 84.5
						Satisfaction: 82.9%		
Rompe	2008	ESWT (low energy)	1	Failed	12	VAS: 7.0 to 3.0	*	
						VISA-A: 53.2 to 79.4	*	
						Satisfaction: 64%		
Saxena	2011	ESWT (low energy)	2	None	12-24	Satisfaction: 82.4%		
Notarnicola	2012	ESWT (low energy)+dietary	1	None	6	VAS: 7.1 to 2.0	*	
						AOFAS: 70.6 to 92.4	*	
						Oximetry: 75.4% to 60.2%	*	
						Satisfaction: 93.8%		
Notarnicola	2012	ESWT (low energy)+placebo	1	None	6	VAS: 7.1 to 2.9	*	
						AOFAS: 65.8 to 76.5	*	
						Oximetry: 73.0% to 66.0%	*	
						Satisfaction: 38.5%		
Taylor	2016	ESWT (low energy)	4	Failed	24	VAS: 6.7 to 2.8	*	
						VISA-A: 43 to 70	*	
Wu	2016	ESWT (low energy)	3	Failed	14.5	VISA-A: 49.6 to 83.9	*	
						Likert: 3.92 to 1.57	*	
Wu	2016	ESWT (low energy) (with Haglund’s deformity)	3	Failed	15.3	VISA-A: 48.7 to 67.8	*	
						Likert: 4.0 to 2.37	*	
Maffulli	2018	ESWT (low energy)	4	Failed	24	VAS: 5.9 to 1.8	*	
						VISA-A: 42.0 to 72.3	*	
						EQ-5D (anxiety): 1.36 to 1.1	n.s.	
						EQ-5D (mobility): 1.71 to 1.35	*	
						EQ-5D (pain): 2.0 to 1.52	*	
						EQ-5D (usual activity): 1.8 to 1.35	*	
						EQ-5D (self-care): 1.11 to 1.0	n.s.	
						EQ-5D (thermometer): 65.3 to 77.3	n.s.	
Pinitkwamdee	2020	ESWT (low energy)+conservative (without ECC)	1	Failed	6	VAS: 6.0 to 2.8	*	
						VAS-FA: 64.8 to 77.2	n.s.	
						Pain: 54.4 to 70.1	n.s.	
						Function: 60.1 to 76.0	n.s.	
						Other complaints: 80.0 to 85.8	n.s.	
Zhang	2020	ESWT (low energy)	3	None	60	VAS: 7.0 to 0.3	*	
						VISA-A: 56 to 90	*	
Zhang	2020	ESWT (low energy)	3	None	60	VAS:7.0 to 1.6	*	
						VISA-A: 51 to 78	*	

*n.s.* no significance

*P < 0.05

### ESWT combined with eccentric training

Five groups received a combined treatment of both ESWT and eccentric training (Table 6). The ESWT in 3 of them applied low-energy shockwave treatments, and the other 2 groups used high-energy shockwave treatments. Three groups had failed in a previous treatment. The VAS scale was evaluated in all 5 patient groups, and the weighted mean of declined value was 4.42 points. VISA-A was assessed in 3 groups, and the mean value increased from 47.9 to 69.4. AOFAS was assessed in 3 groups, and it changed from 68.3 at baseline to 83.4 at the final follow-up. Satisfactory results were recorded in 4 groups, and the total satisfaction rate was 74.3% (84/113).

**Table 6:** Summary of ESWT combined with ECC and outcomes in identified studies

Study	Year	Intervention	LOE	Previous treatment	Follow-up (month)	Evaluation and outcome	Significance	Overall
Notarnicola	2013	ESWT (low energy)+ECC	2	None	6	VAS: 7.0 to 3.3	*	VAS: − 4.42VISA-A: 47.9 to 69.4 Satisfaction: 74.3%(84/113) AOFAS: 68.3 to 93.4
						AOFAS: 67.0 to 76.9	*	
Pavone	2016	ESWT (low energy)+ECC	4	Failed	12	VAS: 7.6 to 1.9	*	
						AOFAS: 71.4 to 91.3	*	
						Satisfaction: 65%		
Erroi	2017	ESWT (low energy)+exercise (include ECC)	3	Failed	6	VAS: 6.4 to 1.5	*	
						VISA-A: 50.6 to 86.5	*	
						Satisfaction: 87.5%		
Wheeler	2019	ESWT (high energy)+exercise (include ECC)	4	NR	6	VAS: 6.5 to 2.0	*	
						VISA-A: 28 to 60	*	
						Satisfaction: 80%		
						Self-reported worst pain: 8.0 to 5.0	*	
						Self-reported stiffness: 6.0 to 3.0	*	
						FAAM-ADL: 57% to 85%	*	
						pD: 15 to 9.5	*	
						EQ-5D(health): 70% to 85%	n.s.	
						HADS (anxiety): 5.0 to 3.0	n.s.	
						HADS (depression): 3.0 to 2.0	n.s.	
Mansur	2019	ESWT (high energy)+ECC	3	NR	6	VAS: 5.3 to 3.2	*	
						VISA-A: 49.1 to 62.6	*	
						AOFAS: 63.6 to 77.2	*	
						Satisfaction: 68.4%		

*n.s.* no significance

*P < 0.05

### Other nonoperative treatments

Other nonoperative treatments were summarized in Table 7. In a pilot study, 11 tendons were injected with sclerosing agent polidocanol, and the VAS scale decreased from 8.3 to 2.8 during 8 months follow-up [11]. Costantino et al. compared three therapies (cryoultrasound therapy, laser therapy CO2, and t.e.ca.r. therapy) in athletes affected by insertional tendonitis and found that every patient benefited from all the treatments [12]. In a prospective case series study, 22 tendons were injected with 25% dextrose-lidocaine. After 28.6 months, the VAS decreased from 7.0 to 1.8 [17]. In a prospective clinical trial, cold air and high-energy laser therapy (CHELT) gave quicker and better pain relief. It also gave the patient a full functional recovery and greater satisfaction [21]. Furia, Kedia and Pinitkwamdee showed that conventional intervention (without ECC) decreased the pain and improved the function [13, 22, 31]. McCormack et al. showed that soft tissue treatment (Astym) plus eccentric exercise improved the function during both short- and long-term follow-up periods [23]. Erroi et al. showed that PRP plus exercise gave a significant improvement of VISA-A and VAS scores at all time-points [27].

**Table 7:** Summary of other treatment and outcomes in identified studies

Study	Year	Intervention	LOE	Previous treatment	Follow-up (month)	Evaluation and outcome	Significance
Öhberg	2003	Sclerosing therapy	4	Failed	8	VAS: 8.3 to 2.8	*
						Satisfaction: 72.7%	
Costantino	2005	Cryoultrasound therapy	3	NR	8	VAS: 9 to 1.8	*
Costantino	2005	Laser CO2	3	NR	8	VAS: 9 to 2.8	*
Costantino	2005	t.e.ca.r. therapy	3	NR	8	VAS: 9 to 2.0	*
Furia	2006	Conservative (without ECC)	3	Failed	12	VAS: 8.6 to 7.0	n.s.
						Satisfaction: 39.4%	
Ryan	2010	Dextrose injections	4	Failed	28.6	VAS at rest: 3.3 to 0.3	*
						VAS with activity: 5.1 to 1.0	*
						VAS during sports: 7.0 to 1.8	*
Notarnicola	2013	CHELT+ECC	2	None	6	VAS: 7.0 to 1.7	*
						AOFAS: 62.5 to 83.0	*
Kedia	2014	Strengthening training: 12 weeks	2	NR	3	VAS: -2.08	*
						SF-36(bodily pain): 16.4	*
						SF-36: 10.27	*
						FAOQ: -0.758	*
McCormack	2016	ECC training+Astym: 12 weeks	2	None	13	VISA-A: 36.6 to 90.7	*
						NPRS: 4.6 to 0.67	*
						Satisfaction: 100%	
Erroi	2017	PRP+exercise (include ECC)	3	Failed	6	VAS: 5.9 to 2.6	*
						VISA-A: 52.8 to 82.0	*
						Satisfaction: 71.4%	
Pinitkwamdee	2020	Conservative (without ECC)	1	Failed	6	VAS: 5.2 to 2.0	*
						VAS-FA: 65.3 to 82.7	n.s.
						Pain: 47.3 to 77.8	*
						Function: 66.6 to 82.5	n.s.
						Other complaints: 83.9 to 87.9	n.s.

*n.s.* no significance

*P < 0.05

### Comparative studies

There were 11 comparative studies (5 retrospective cohort studies, 6 RCTs) with regard to different interventions (Table 8). Costantino et al. compared three therapies (cryoultrasound therapy, laser therapy CO2, and t.e.ca.r. therapy) in athletes affected by insertional tendonitis and found that cryoultrasound showed significant advantages over the other two, but there was no significant difference between laser therapy CO2 and t.e.ca.r. therapy [12]. Furia et al. showed that high-energy ESWT was more effective than conventional nonoperative treatment (rest, medication, activity modification, stretching exercise, and heel lift orthosis) whenever VAS or satisfaction rates were evaluated [13]. In a RCT from Rompe et al., eccentric loading showed inferior results to low-energy ESWT as applied in patients with chronic recalcitrant insertional tendinopathy at 4 months of follow-up [15]. Notarnicola et al. demonstrated that ESWT with additional dietary supplement containing arginine, Vinitrox™, collagen, methyl-sulfonyl-methane, vitamin C, and bromelain significantly improved the therapeutic response when compared to ESWT with placebo [20]. In another RCT from Notarnicola et al., high-energy laser therapy gave quicker pain relief and gave the patients greater satisfaction than low-energy ESWT [21]. McCormack et al. found that soft tissue treatment (Astym) plus eccentric training was more effective than eccentric exercise alone at improving function (VISA-A) during both short- and long-term follow-up periods [23]. However, Kedia et al. found that conventional physical therapy consisting of gastrocnemius, soleus and hamstring stretches, ice, and use of heel lifts and night splints with or without eccentric training were both effective and showed no significant difference [22]. Besides, no difference was found at 24 weeks between the standard nonoperative treatment combined with low-energy ESWT and low-energy ESWT alone for chronic insertional Achilles tendinopathy, especially in elderly patients [31]. Wu et al. found that ESWT resulted in greater clinical outcomes in patients without Haglund’s deformity compared with patients with Haglund’s deformity [26]. Erroi et al. demonstrated that both ESWT and PRP therapy were effective and safe, and there were no significant differences between two groups in VAS, VISA-A, or satisfaction rate [27]. Zhang et al. showed that patients with IAT who had greater sports activity levels had better therapeutic responses to ESWT than nonsports-active patients after a 5-year follow-up [32].

**Table 8:** Summary of comparative studies

Study	Year	Intervention	LOE	Previous treatment	Follow-up (month)	Evaluation and outcome	Significance
Costantino	2005	Cryoultrasound therapy vs. laser CO2 vs. t.e.ca.r. therapy	3	NR	8	VAS: 1.8 vs. 2.8 vs. 2.0	n.s.
Furia	2006	ESWT (high energy) vs. conservative (without ECC)	3	Failed	12	VAS: 2.8 vs. 7.0	*
						Satisfaction: 82.9% vs. 39.4%	*
Rompe	2008	ESWT (low energy) vs. ECC training (full range)	1	Failed	12	VAS: 3.0 vs. 5.0	*
						VISA-A: 79.4 vs. 63.4	*
						Satisfaction: 64% vs. 28%	*
Notarnicola	2012	ESWT (low energy)+dietary vs. ESWT (low energy)+placebo	1	NR	6	VAS: 2.0 vs. 2.9	n.s.
						AOFAS: 92.4 vs. 76.5	*
						Oximetry: 60.2% vs. 66.0%	*
						Satisfaction: 93.8% vs.38.5%	*
Notarnicola	2013	CHELT+ECC vs. ESWT (low energy)+ECC	2	None	6	VAS: 1.7 vs. 3.3	*
						AOFAS: 83.0 vs. 76.9	n.s.
Kedia	2014	ECC (full range)+strengthening training vs. strengthening training	2	NR	3	VAS: − 2.19 vs. − 2.08	n.s.
						SF-36 (bodily pain): 16.22 vs. 16.4	n.s.
						SF-36: 9.78 vs. 10.27	n.s.
						FAOQ: − 0.73 vs. − 0.758	n.s.
McCormack	2016	ECC training+Astym vs. ECC training	2	None	3	VISA-A: 67.0 vs. 90.7	*
						NPRS: 1.0 vs. 0.67	n.s.
						Satisfaction: 83.3% vs. 100%	n.s.
Wu	2016	ESWT (low energy) vs. ESWT (low energy) (with Haglund’s deformity)	3	Failed	14.5	VISA-A: 83.9 vs. 67.8	*
						Likert: 1.57 vs. 2.37	n.s.
Erroi	2017	ESWT (low energy)+exercise (include ECC) vs. PRP+exercise (include ECC)	3	Failed	6	VAS: 1.5 vs. 2.6	n.s.
						VISA-A: 86.5 vs. 82.0	n.s.
						Satisfaction: 87.5% vs. 71.4%	n.s.
Pinitkwamdee	2020	ESWT (low energy)+conservative (without ECC) vs. conservative (without ECC)	1	Failed	6	VAS: 2.8 vs. 2.0	n.s.
						VAS-FA: 77.2 vs. 82.7	n.s.
						Pain: 70.1 vs. 77.8	n.s.
						Function: 76.0 vs. 82.5	n.s.
						Other complaints: 85.8 vs. 87.9	n.s.
Zhang	2020	ESWT (low energy) (sports-active) vs. ESWT (low energy) (nonsports-active)	3	None	60	VAS: 0.3 to 1.6	*
						VISA-A: 90 to 78	*

*n.s.* no significance

*P < 0.05

## Discussion

### Eccentric exercises

Eccentric exercises are traditional and one of the most recommended nonoperative treatments for Achilles tendinopathy [33–35], especially for mid-portion/non-insertional Achilles tendinopathy [34, 36]. Eccentric exercises were reported to decrease local pain by strengthening the calf muscle, lengthening of the myotendinous unit, and decreasing neovascularization in the region. The combined results of 6 groups in this systematic review showed that the weighted mean of VAS scale had a decline of 2.83 points, which might suggest that eccentric training was an effective treatment for IAT. However, there were no RCTs to compare the eccentric training with sham control to make a firm conclusion. Besides, a prospective cohort study from Fahlström et al. found that eccentric training resulted in only 32% satisfaction in patients with IAT, while the rate (89%) was much higher in patients with mid-portion Achilles tendinopathy [6]. In a RCT, conventional physical therapy with or without eccentric training exerted equal effects for IAT [22]. So, whether eccentric exercises are suitable for IAT is still open for debate. Because of the limited and conflicting evidence, eccentric exercise was given a Grade I recommendation according to the Grades of Recommendation [2]. Further RCTs of large samples comparing eccentric exercises and sham control (wait and see) groups are needed. Moreover, maximum load, speed of contraction, and frequency of sessions should also be studied and optimized.

### Extracorporeal shock wave therapy

Recently, high- and low-energy ESWT have been used for the treatment of Achilles tendinopathy and have shown good outcomes [13, 28, 31, 37]. The weighted mean of decreased VAS in 9 of 12 groups was 4.49 points (larger than that of eccentric exercises) at the final follow-up. The overall satisfaction rate of ESWT of the included studies is 73.7%, much higher than that of eccentric training (45.6%). Among the total 11 groups, patients from 6 groups, who were enrolled in these studies to receive ESWT, had unsatisfactory results from other nonoperative treatments before. Besides, in a RCT reported by Rompe et al., low-energy ESWT showed superiority over eccentric training in patients with chronic recalcitrant tendinopathy at the 4-month follow-up [15]. In a retrospective comparative study, high-energy ESWT was more effective than traditional nonoperative methods after the 12-month follow-up [13]. However, another RCT indicated that low-energy ESWT had no significant benefit for IAT at the 24 weeks follow-up, especially in the elderly [31]. But the sample size (16/15) was small, and the younger patients were not fully evaluated. One big problem of ESWT is the high amount of pain during the treatment process. Whether local anesthesia field block should be used and whether anesthesia would affect the outcome remain open for debate. Overall, ESWT is widely used and supported now and has a Grade B recommendation. Further RCTs with a large sample size are needed to verify the effectiveness more accurately.

### Injection therapy

The current therapy of medicinal injections for treating Achilles tendinopathy included sclerosing therapy, hyperosmolar dextrose injections, corticosteroid injections, and platelet-rich plasma (PRP) injections. However, studies with high-quality evidence are rare, especially for insertional form of Achilles tendinopathy. It was considered that the pain was due to the neovascularization outside and inside the affected tendon [38] and could be alleviated in most patients if the neovessels were destroyed by injections of a sclerosing agent [39]. In a pilot study in 2003, the injections of sclerosing agent polidocanol against the local neovessels relieved the pain in 8 out of 11 patients with IAT at the 8-month follow-up [11]. Dextrose, as a kind of prolotherapy, is considered to elicit a proliferant cellular response by inducing inflammation, subsequent growth factor production leading to increased fibroblast proliferation (either locally or systemic) and increased production of extracellular matrix materials [40]. In a retrospective case series report, dextrose injections reduced pain from either the insertion or mid-portion Achilles tendinopathy at the 28.6 months follow-up [17]. Platelet-rich plasma (PRP) injections are a regenerative treatment for Achilles tendinopathy, and its mechanism in vivo is still unclear. Up to date, there was only one study reporting PRP injections in treatment of specific IAT. In this retrospective study, a combination of PRP injections and eccentric exercises was equally effective to combining treatment of ESWT and eccentric training after the 6-month follow-up [27]. Corticosteroid injections have largely fallen out of clinical practice for tendinopathy treatment for the risk of tendon degeneration and tear, and no studies were found applying corticosteroid injection for IAT [41]. Overall, studies regarding various injection therapy for IAT are rare and more evidence are required (Grade I recommendation).

### Combination treatment

IAT is considered to be more refractory than mid-portion AT, so more trials focus on a combination treatment. In this review, 5 groups were treated with a combination of ESWT and eccentric exercises, which were two of the most widely used nonoperative therapies. The weighted mean of declined VAS value was 4.42 points. The total satisfaction rate in 4 groups was 74.3%. These results were similar to those of cases that received single ESWT (73.7%), but much higher than those of eccentric exercises alone (45.6%). However, RCTs of direct comparison between the combination and either treatment were lacking, and the superiority of this combination treatment could not be confirmed. Other combination treatments, including Notarnicola et al. (high-energy laser therapy and eccentric exercises) [21], McCormack et al. (soft tissue treatment and eccentric exercise) [23] and Erroi et al. (PRP and home exercises) [27], were all suggested to be effective, but they were all reported by individual study and lacked generalized evidence. In summary, the combination treatment (ESWT and eccentric exercises) for IAT has a Grade B recommendation, and other combination treatments have a Grade I recommendation.

### Limitations

One of the main limitations of this review is the low level of evidence and risk of bias of the included studies. Only 6 studies were RCTs. For many studies, the outcome of treatment is evaluated by a patient-based self-comparison (pre- vs. post- therapy), and a sham/control (wait and see) group is needed to reflect the effectiveness authentically and accurately. Secondly, the terminology of Achilles tendon pathology varies among studies, so some studies may be excluded during the process of literature screening according to our strict inclusion criteria. Thirdly, for some studies, the diagnosis is based on clinical findings alone without imaging confirmation, which may enlarge the scope of included cases or add the risk of bias.

## Conclusion

IAT is regarded as a distinct clinical entity which is often accompanied with metabolic diseases and is difficult to manage, and the treatment and prognosis of IAT are not totally the same to those of mid-portion/non-insertional Achilles tendinopathy. Although more evidence is in support of eccentric exercises than the other interventions for mid-portion/non-insertional Achilles tendinopathy, eccentric exercises did not result in a high satisfaction rate for IAT. Current evidence for nonoperative treatment favors ESWT or the combined treatment of ESWT plus eccentric exercises. Evidence in support of other therapies (including sclerosing therapy, dextrose injections, strengthening training, PRP, soft tissue treatment) are lacking, and more investigation with high level of evidence is needed.

## Supplementary Information

**Additional file 1.** PRISMA 2009 checklist.

## Data Availability

The datasets used and/or analyzed during the current study are available from the corresponding author on reasonable request.
